# Joint trajectories of loneliness and depressive symptoms and risk of incident osteoporosis among middle-aged and older adults: a prospective cohort study

**DOI:** 10.3389/fendo.2026.1866762

**Published:** 2026-09-03

**Authors:** Dongnan Chen, Lincheng Duan, Xiaoxue Huang, Jingyu Li, Dan Wu

**Affiliations:** 1 Department of Traditional Chinese Medicine, Fengjie Hospital Affiliated to Chongqing Three Gorges Medical College, Chongqing, China; 2 Chengdu University of Traditional Chinese Medicine, Chengdu, China

**Keywords:** depressive symptoms, group-based multi-trajectory modelling, joint trajectories, loneliness, older adults, osteoporosis

## Abstract

**Background:**

Loneliness, depressive symptoms, and osteoporosis are major public health concerns in ageing populations worldwide. Although the interplay between mental and physical health is well recognized, evidence remains limited regarding whether long-term joint patterns of loneliness and depressive symptoms are prospectively associated with incident osteoporosis. This study aimed to investigate the prospective associations between joint trajectories of loneliness and depressive symptoms and incident osteoporosis in middle-aged and older adults using a nationally representative cohort.

**Methods:**

Data were obtained from the English Longitudinal Study of Ageing (ELSA). A total of 4,117 participants aged ≥50 years who were free of osteoporosis at Wave 4 were included. Group-based multi-trajectory modelling was used to identify joint trajectories of loneliness, assessed by the UCLA Loneliness Scale, and depressive symptoms, assessed by the Center for Epidemiologic Studies Depression Scale (CES-D), across Waves 2 to 4. Incident osteoporosis was defined as self-reported physician-diagnosed osteoporosis during follow-up from Waves 5 to 9. Cox proportional hazards models were used to examine the associations between trajectory group membership and the risk of incident osteoporosis after adjusting for sociodemographic characteristics, health status, and lifestyle factors.

**Results:**

Three distinct joint trajectories of loneliness and depressive symptoms were identified: a low loneliness/low depressive symptoms stable group (70.83%), a moderate loneliness/low depressive symptoms group (21.54%), and a persistent high loneliness/high depressive symptoms group (7.63%). During follow-up, 276 participants developed osteoporosis. In the fully adjusted model, compared with the low loneliness/low depressive symptoms stable group, participants in the persistent high loneliness/high depressive symptoms group had a significantly higher risk of incident osteoporosis (HR = 1.97, 95% CI: 1.37–2.83; *P* < 0.001). Participants in the moderate loneliness/low depressive symptoms group also had an increased risk of osteoporosis (HR = 1.58, 95% CI: 1.19–2.10; *P* = 0.002).

**Conclusion:**

Persistent loneliness and depressive symptoms were associated with a higher risk of incident osteoporosis among middle-aged and older adults. These findings indicate that psychological well-being may be relevant to osteoporosis risk assessment and healthy ageing, while not establishing a causal relationship.

## 1 Introduction

With the rapid ageing of the global population, age-related chronic diseases have become a major public health challenge (1). Osteoporosis is one of the most common metabolic bone diseases in older adults. Its resulting fragility fractures, particularly hip fractures, are closely associated with substantial functional decline, reduced quality of life, premature mortality, and a considerable burden on healthcare systems (2). Although traditional risk factors for osteoporosis, such as older age, female sex, low body weight, physical inactivity, and medication use, have been extensively studied (3), its psychosocial determinants remain relatively underexplored.

Loneliness and depressive symptoms are highly prevalent and increasingly recognized mental health problems in older populations. Studies and systematic reviews have shown that social isolation, loneliness, and depression are associated with a higher risk of cardiovascular disease (4, 5), cognitive decline (6, 7), and all-cause mortality (5, 8). The magnitudes of these reported associations may be comparable with those of conventional biomedical risk factors (9). Following the COVID-19 pandemic, the public health significance of prolonged social disconnection and psychological distress among older adults has become even more apparent (10), highlighting the need to characterize their associations with physical health.

Emerging evidence suggests an important, yet insufficiently understood, connection between mental health and skeletal health (11). Previous studies have reported that individuals with depression tend to have lower bone mineral density and a higher risk of fractures (12–14). However, prospective evidence remains limited regarding whether more common psychological conditions in the general community, particularly loneliness and depressive symptoms, are associated with incident osteoporosis. This gap is especially notable when distinguishing transient distress from long-term and persistent psychological exposure (15, 16).

Several biological and behavioral pathways may be relevant to the association between loneliness, depression, and bone health. Osteoporosis is multifactorial and is not assumed to be driven primarily by inflammation in this context. Chronic psychological stress has been associated with hypothalamic-pituitary-adrenal-axis dysregulation and greater cumulative glucocorticoid exposure; these processes may suppress bone formation and favor bone resorption (17–19). In addition, the low-grade inflammatory state associated with depression may be linked to bone loss through proinflammatory cytokine-mediated disturbances in bone remodeling (20). Psychological distress also commonly co-occurs with reduced physical activity, smoking, alcohol consumption, poor nutrition, and vitamin D deficiency, which are themselves associated with poorer skeletal health (21, 22). These pathways provide biological plausibility but were not tested directly in this study.

Notably, most previous studies used single-time-point assessments of mental health and therefore were unable to capture its dynamic changes over time (13, 23, 24). Yet loneliness and depressive symptoms are heterogeneous across the life course, and distinct long-term trajectories, such as persistently low levels, chronically high levels, or progressive worsening, may show different associations with health outcomes (25, 26). Trajectory modelling provides a more refined framework for identifying subgroups with similar developmental patterns and for evaluating associations with cumulative psychological burden (27–29). Group-based multi-trajectory modelling (GBMTM) is a finite-mixture approach that identifies subgroups with similar joint longitudinal patterns. In this study, GBMTM was used to summarize the cumulative pre-index pattern of loneliness and depressive symptoms; analyzing the repeated measures as time-varying exposures would instead estimate associations with updated, more proximal values and answer a complementary question. However, this trajectory approach has rarely been applied in osteoporosis research.

Therefore, using data from the ELSA, a nationally representative prospective cohort, we characterized joint trajectories of loneliness and depressive symptoms across Waves 2 to 4 among middle-aged and older adults (MAOAs) and examined their associations with the subsequent risk of incident osteoporosis. We hypothesized that, compared with individuals with persistently low levels of loneliness and depressive symptoms, those with persistent or worsening psychological distress would have a higher risk of developing osteoporosis, even after considering sociodemographic characteristics, health status, and lifestyle-related factors.

## 2 Methods

### 2.1 Study design and population

This population-based prospective cohort study used data from the ELSA (30). ELSA is an ongoing, nationally representative, multidisciplinary study created to investigate the health, social circumstances, wellbeing, as well as economic conditions of MAOAs living in England. The study began in 2002, with follow-up assessments conducted approximately every 2 years thereafter. Detailed information on the study design as well as sampling procedures has been published elsewhere. The National Research Ethics Service granted ELSA ethical permission, and each participant gave signed informed consent.

**Figure 1:**
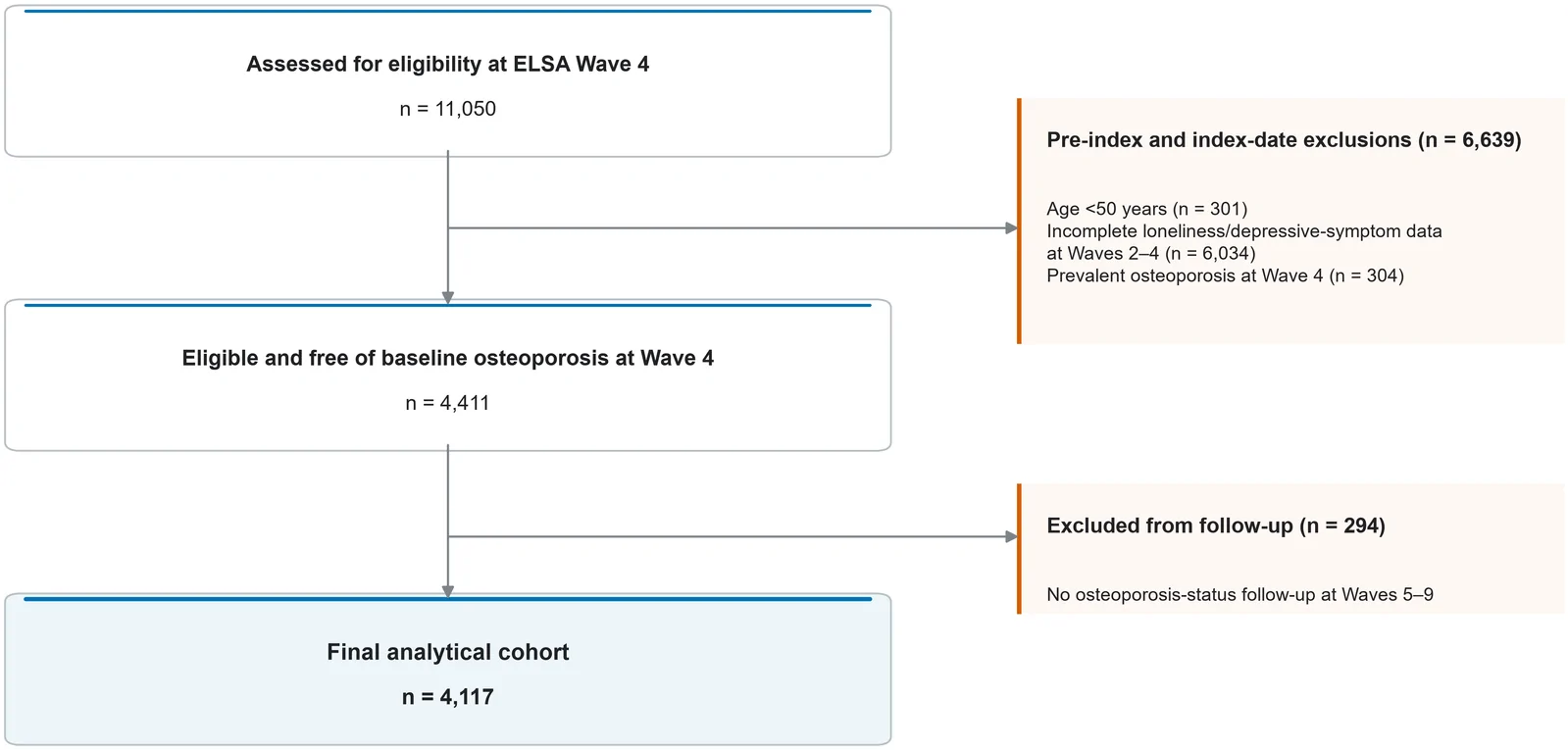
Participant flowchart. Participants were assessed for eligibility at ELSA Wave 4; exclusions were applied before the outcome follow-up. ELSA, English Longitudinal Study of Ageing.

**Figure 2:**
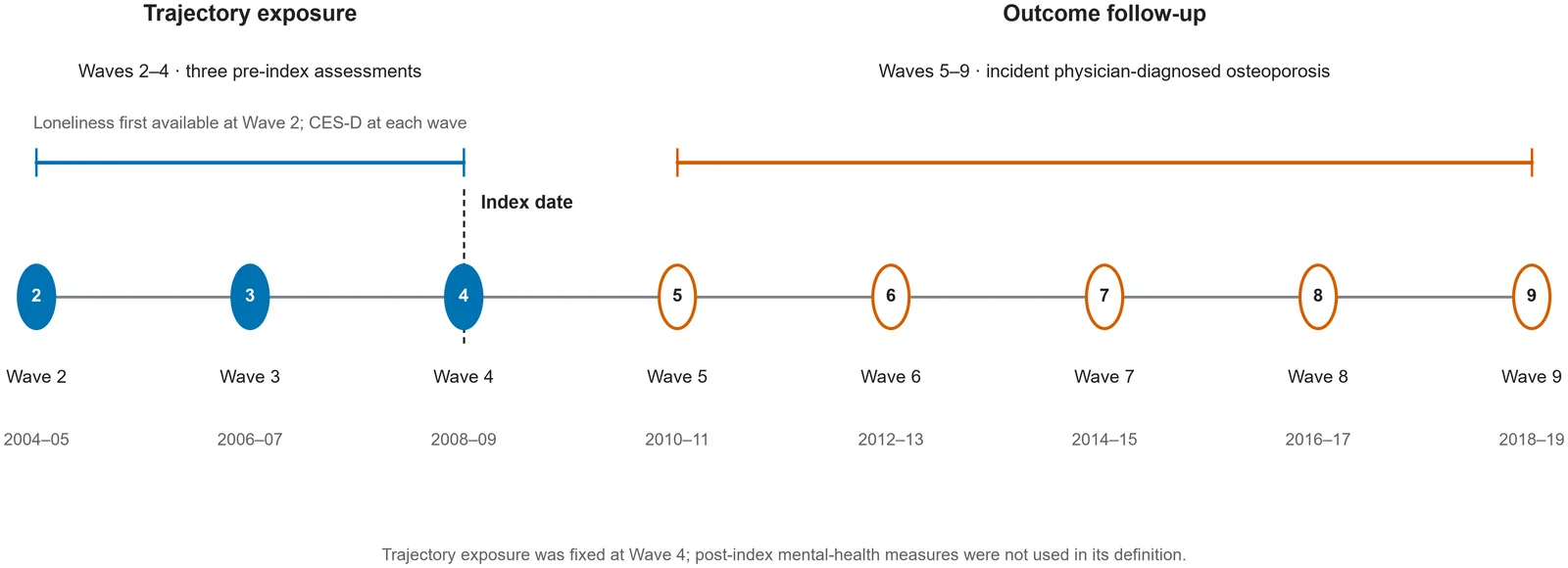
Time frame for the joint trajectories of loneliness and depressive symptoms and the subsequent osteoporosis follow-up. Loneliness was first assessed at Wave 2; Waves 2–4 were used for trajectory modelling, Wave 4 served as the index date, and incident osteoporosis was assessed during Waves 5–9.

In this study, data from Waves 2 to 9 of ELSA were used. Loneliness was first assessed at Wave 2; therefore, the earliest three consecutive assessments (Waves 2 to 4) were used to construct the joint trajectories. Three repeated observations provided the minimum longitudinal information required for the trajectory modelling applied here. Wave 4 was treated as the baseline for survival analysis, and incident osteoporosis was identified during follow-up from Waves 5 to 9. This choice ensured that all trajectory measurements preceded the outcome-risk period and retained the longest subsequent follow-up. Incorporating later mental-health assessments would either require a later baseline and shorter follow-up or allow exposure measurements to overlap with osteoporosis onset. Participants were eligible if they were aged ≥50 years, free of osteoporosis at Wave 4, and had complete loneliness and depressive-symptom data across Waves 2 to 4. Individuals without follow-up information on osteoporosis status were excluded. Detailed information on systemic glucocorticoids, bisphosphonates, and other bone-active medications was not available in the analytic dataset; participants were therefore not excluded on the basis of these medications. After applying these criteria, 4,117 participants were included in the final analysis (**Figures 1**, **2**).

### 2.2 Exposure assessment

Loneliness was assessed using the 3-item version of the University of California, Los Angeles (UCLA) Loneliness Scale (31–33). Participants were asked how often they felt lacking companionship, left out, and isolated from others. Response options for each item were “hardly ever or never” (1 point), “some of the time” (2 points), and “often” (3 points). Total scores ranged from 3 to 9, with higher scores indicating greater loneliness. The UCLA-3 was developed for population surveys and has demonstrated acceptable reliability and convergent validity (32); cross-country analyses using ELSA and the US National Social Life, Health, and Aging Project have also examined differential item functioning of its items (33).

Depressive symptoms were measured using the 8-item CES-D (34). Total scores ranged from 0 to 8, with higher scores indicating more severe depressive symptoms. Longitudinal evaluation in ELSA supports the reliability and measurement invariance of the CES-D-8 across time and sex (35).

Both loneliness and depressive symptoms were repeatedly assessed at Waves 2 to 4, providing the longitudinal data used for trajectory modelling. The trajectory labels were descriptive summaries of the fitted score patterns rather than clinical cut-off categories.

### 2.3 Outcome

The outcome of interest was incident osteoporosis. During follow-up from Waves 5 to 9, participants were classified as having incident osteoporosis if they reported that a doctor had diagnosed them with osteoporosis. Follow-up time was calculated from the baseline assessment (Wave 4) until the first report of osteoporosis or the end of follow-up at Wave 9, whichever came first.

### 2.4 Covariates

To control for potential confounding, a range of covariates measured at baseline (Wave 4) were included in the analysis; the proportions of missing data are displayed in **Supplementary Table S1**. These covariates included:

Sociodemographic characteristics: age (continuous), sex, ethnicity (White/non-White), and marital status (married/other). The other category comprised participants who were cohabiting, divorced, never married, separated, or widowed.

Socioeconomic status: educational attainment (below high school, high school, or college or above) and household wealth, categorized into sample-specific quintiles from Q1 (lowest) to Q5 (highest), with Q1 as the reference category. No external fixed cut-offs were used for the wealth quintiles.

Health behaviors: physical activity level (low/high), smoking status (yes/no), and alcohol consumption frequency (≥1 time/week vs. <1 time/week). Physical activity was classified as high if participants reported moderate- or vigorous-intensity activity at least once per week; otherwise, it was classified as low.

Health status: body mass index (BMI), calculated as weight in kilograms divided by height in meters squared (kg/m²) and included as a continuous variable, and baseline diabetes, hyperlipidemia, cancer, and hypertension (yes/no).

### 2.5 Statistical analysis

#### 2.5.1 Identification of joint trajectories

Group-based multi-trajectory modelling (GBMTM) was used to jointly model loneliness scores and CES-D depressive-symptom scores across Waves 2 to 4 and to identify latent subgroups with distinct joint patterns (28). GBMTM is a finite-mixture approach in which participants are assigned to the class with the highest posterior probability. Candidate models M1–M9 included two-, three-, and four-class solutions with linear, quadratic, or cubic polynomial terms for both outcomes; for example, [111][111] denotes three classes with a linear term for each outcome in each class. Model selection was based jointly on the Bayesian information criterion (BIC), entropy, average posterior probabilities (APPs), odds of correct classification (OCC), minimum group size (>5%), parsimony, and substantive interpretability rather than on BIC alone. BIC became progressively less negative as the number of classes increased. The three-class linear model (M4) retained strong classification performance, with a log likelihood of −40,927.969, BIC of −40,994.552, AIC of −40,943.969, entropy of 0.910, class-specific APPs of 91.205%, 97.409%, and 94.152%, OCC values exceeding 15, and a minimum observed class proportion of 7.63%. Quadratic and cubic specifications provided negligible gains in entropy and included unstable higher-order estimates. The four-class linear model (M7) also showed acceptable classification performance (entropy, 0.913; minimum APP, 85.271%; minimum observed class proportion, 7.26%), but it primarily subdivided the intermediate pattern into two smaller phenotypes while retaining the low/stable and high/high patterns. To avoid overfragmentation and preserve a parsimonious, interpretable primary exposure classification, M4 was selected for the primary analysis; M7 was evaluated as a sensitivity analysis rather than discarded.

#### 2.5.2 Main analysis

Baseline characteristics were summarized using descriptive statistics. As appropriate, variables were presented as frequency (%), median (interquartile range), or mean ± SD. The chi-square test was used to compare categorical variables, while one-way analysis of variance (ANOVA) or the Kruskal-Wallis test was used to compare continuous variables.

Cox proportional hazards regression models were used to examine the associations between joint loneliness-depressive symptom trajectories and incident osteoporosis, with results reported as HRs and 95% CIs. The proportional hazards assumption was assessed using Schoenfeld residuals. The low loneliness/low depressive symptoms stable group was used as the reference category.

Three progressively adjusted models were constructed: Model 1 was unadjusted; Model 2 was adjusted for age, sex, ethnicity, marital status, education, and household wealth; and Model 3 additionally included BMI, diabetes, hyperlipidemia, cancer, hypertension, smoking, alcohol consumption, and physical activity. Subgroup analyses were conducted by age, sex, marital status, education, BMI, smoking, alcohol consumption, and physical activity. Sensitivity analyses used multivariable logistic regression, excluded osteoporosis events at the first follow-up wave, and repeated the analysis using the four-class trajectory solution.

SAS version 9.4 and R version 4.4.3 were used for all analyses, and two-sided P < 0.05 was considered statistically significant.

## 3 Results

### 3.1 Baseline characteristics

**Figure 3:**
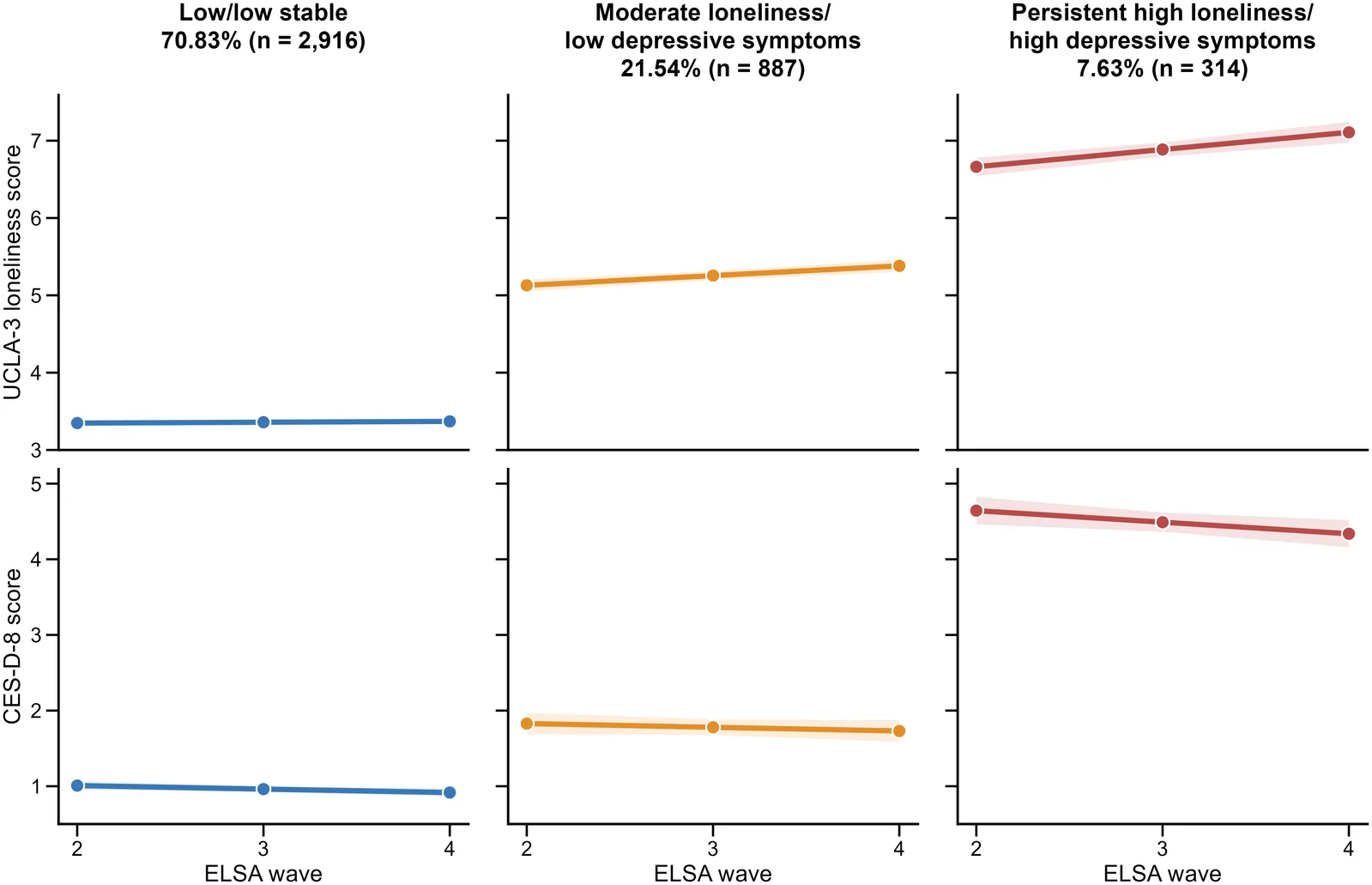
Joint trajectories of loneliness and depressive symptoms across Waves 2 to 4. Trajectory names describe the fitted patterns and do not represent clinical categories. CES-D-8, 8-item Center for Epidemiologic Studies Depression Scale; UCLA-3, 3-item UCLA Loneliness Scale.

A total of 4,117 participants were included, with a mean age of 67.43 ± 8.56 years, and 45.08% were men. During a mean follow-up of 98.22 ± 33.17 months, 276 incident cases of osteoporosis were documented (**Table 1**). The group-based multi-trajectory model identified three joint trajectories of loneliness and depressive symptoms: a low loneliness/low depressive symptoms stable group (70.83%; n = 2,916), a moderate loneliness/low depressive symptoms group (21.54%; n = 887), and a persistent high loneliness/high depressive symptoms group (7.63%; n = 314). The fitted moderate/low trajectory began at a moderate loneliness level and increased modestly, while depressive symptoms remained low and declined slightly. The persistent high/high trajectory had the highest fitted scores for both outcomes across Waves 2 to 4, although depressive symptoms declined slightly. Detailed model-fitting information is presented in **Supplementary Tables S2*****–*****S5**; **Figure 3**.

**Table 1:** Baseline characteristics of the study population by joint trajectory of loneliness and depressive symptoms.

Characteristic	Total (n = 4,117)	Low/low stable (n = 2,916)	Moderate/low (n = 887)	Persistent high/high (n = 314)	Statistic	*P*
Age, mean ± SD	67.43 ± 8.56	67.21 ± 8.37	67.77 ± 8.97	68.54 ± 9.01	*F* = 4.32	0.013
BMI, mean ± SD	28.42 ± 5.22	28.31 ± 5.08	28.61 ± 5.51	28.86 ± 5.59	F=2.28	0.102
Follow-up (months), mean ± SD	98.22 ± 33.17	100.20 ± 32.15	94.80 ± 34.42	89.49 ± 36.67	F=20.98	<.001
Sex, n (%)					χ²=60.83	<.001
Female	2261 (54.92)	1504 (51.58)	529 (59.64)	228 (72.61)		
Male	1856 (45.08)	1412 (48.42)	358 (40.36)	86 (27.39)		
Ethnicity, n (%)					χ²=1.37	0.503
Non-white	43 (1.04)	27 (0.93)	12 (1.35)	4 (1.27)		
White	4074 (98.96)	2889 (99.07)	875 (98.65)	310 (98.73)		
Marital status, n (%)					χ²=364.98	<.001
Married	2922 (70.97)	2310 (79.22)	493 (55.58)	119 (37.90)		
Other	1195 (29.03)	606 (20.78)	394 (44.42)	195 (62.10)		
Education, n (%)					χ²=48.45	<.001
Below high school	1494 (36.29)	984 (33.74)	357 (40.25)	153 (48.73)		
High school	928 (22.54)	643 (22.05)	218 (24.58)	67 (21.34)		
College or above	1695 (41.17)	1289 (44.20)	312 (35.17)	94 (29.94)		
Household wealth, n (%)					χ²=77.63	<.001
Q1	824 (20.01)	599 (20.54)	165 (18.60)	60 (19.11)		
Q2	823 (19.99)	540 (18.52)	206 (23.22)	77 (24.52)		
Q3	823 (19.99)	540 (18.52)	204 (23.00)	79 (25.16)		
Q4	823 (19.99)	569 (19.51)	177 (19.95)	77 (24.52)		
Q5	824 (20.01)	668 (22.91)	135 (15.22)	21 (6.69)		
Current smoking, n (%)					χ²=30.46	<.001
No	3674 (89.24)	2639 (90.50)	782 (88.16)	253 (80.57)		
Yes	443 (10.76)	277 (9.50)	105 (11.84)	61 (19.43)		
Alcohol consumption, n (%)					χ²=76.41	<.001
<1/week	1520 (36.92)	966 (33.13)	381 (42.95)	173 (55.10)		
≥1/week	2597 (63.08)	1950 (66.87)	506 (57.05)	141 (44.90)		
Physical activity, n (%)					χ²=141.63	<.001
High	3347 (81.30)	2482 (85.12)	679 (76.55)	186 (59.24)		
Low	770 (18.70)	434 (14.88)	208 (23.45)	128 (40.76)		
Diabetes, n (%)					χ²=13.52	0.001
No	3628 (88.12)	2603 (89.27)	762 (85.91)	263 (83.76)		
Yes	489 (11.88)	313 (10.73)	125 (14.09)	51 (16.24)		
Hyperlipidemia, n (%)					χ²=25.15	<.001
No	2685 (65.22)	1967 (67.46)	543 (61.22)	175 (55.73)		
Yes	1432 (34.78)	949 (32.54)	344 (38.78)	139 (44.27)		
Cancer, n (%)					χ²=3.32	0.190
No	3771 (91.60)	2678 (91.84)	814 (91.77)	279 (88.85)		
Yes	346 (8.40)	238 (8.16)	73 (8.23)	35 (11.15)		
Hypertension, n (%)					χ²=2.23	0.328
No	1791 (43.50)	1285 (44.07)	381 (42.95)	125 (39.81)		
Yes	2326 (56.50)	1631 (55.93)	506 (57.05)	189 (60.19)		
Incident osteoporosis, n (%)					χ²=39.71	<.001
No	3841 (93.30)	2761 (94.68)	809 (91.21)	271 (86.31)		
Yes	276 (6.70)	155 (5.32)	78 (8.79)	43 (13.69)		

Values are mean ± SD or n (%). F, one-way analysis of variance; χ², chi-square test; BMI, body mass index; SD, standard deviation.

### 3.2 Associations of joint trajectories of loneliness and depressive symptoms with incident osteoporosis

**Table 2** presents the associations between joint trajectories of loneliness and depressive symptoms and the risk of incident osteoporosis. Compared with the low loneliness/low depressive symptoms stable group, the moderate loneliness/low depressive symptoms group and persistent high loneliness/high depressive symptoms group were both significantly associated with an increased risk of osteoporosis.

**Table 2:** Associations between joint trajectories of loneliness and depressive symptoms and the risk of incident osteoporosis.

Joint trajectory	Model 1 HR (95% CI)	*P*	Model 2 HR (95% CI)	*P*	Model 3 HR (95% CI)	*P*
Low/low stable	1.00 (reference)		1.00 (reference)		1.00 (reference)	
Moderate/low	1.77 (1.35–2.33)	<0.001	1.51 (1.14–2.01)	0.004	1.58 (1.19–2.10)	0.002
Persistent high/high	2.95 (2.10–4.13)	<0.001	2.00 (1.40–2.85)	<0.001	1.97 (1.37–2.83)	<0.001
HR, hazard ratio; CI, confidence interval.						

Model 1: unadjusted. Model 2: adjusted for age, sex, ethnicity, marital status, education, and household wealth. Model 3: additionally adjusted for BMI, diabetes, hyperlipidemia, cancer, hypertension, current smoking, alcohol consumption, and physical activity.

**Figure 4:**
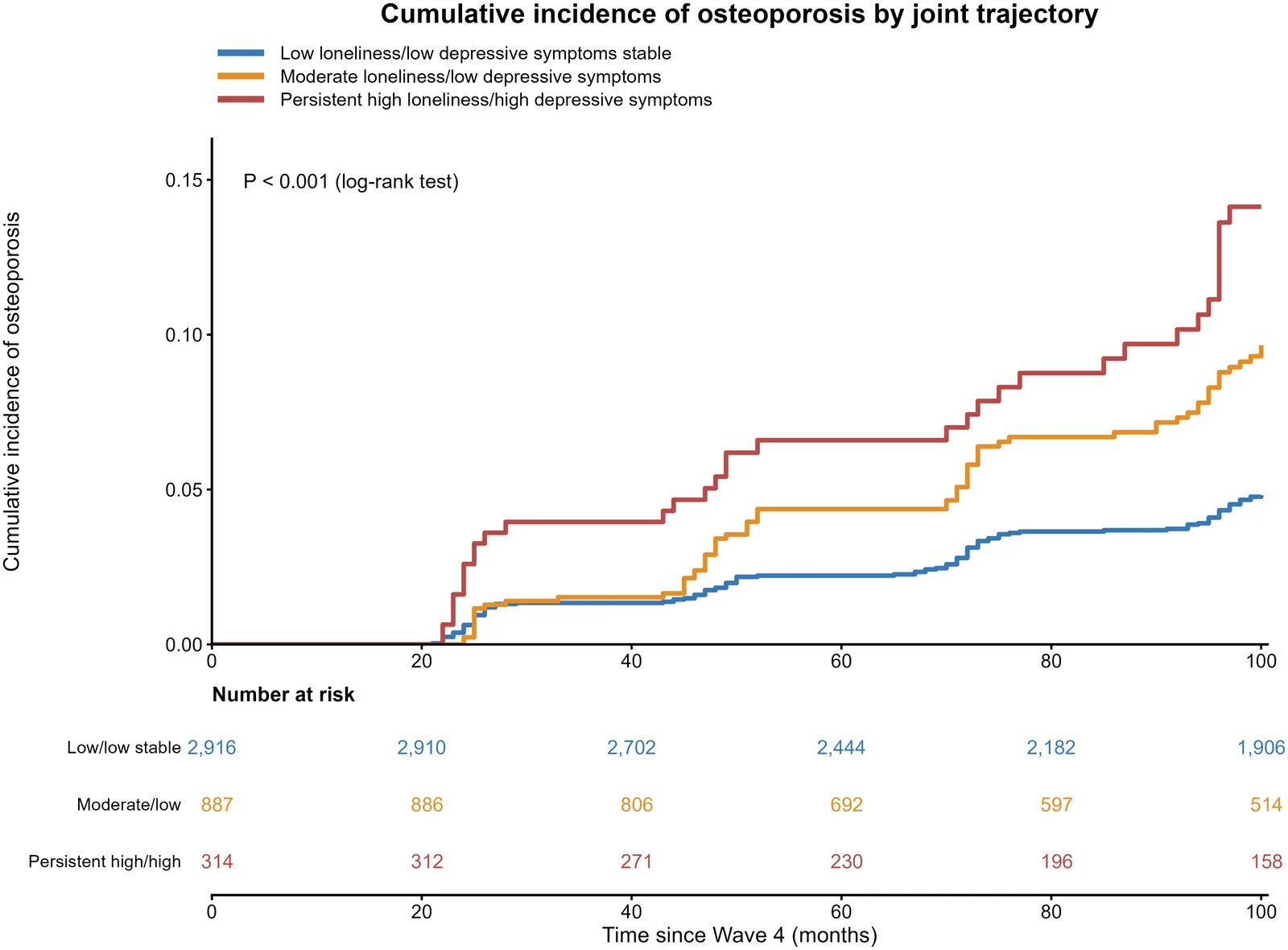
Kaplan–Meier curves for the cumulative incidence of osteoporosis by joint trajectory of loneliness and depressive symptoms. The risk table shows the number of participants remaining under observation. The P value is from a two-sided log-rank test.

In the fully adjusted model, compared with the reference group, the risk of incident osteoporosis was 58% higher in the moderate loneliness/low depressive symptoms group (HR = 1.58, 95% CI: 1.19–2.10; *P* = 0.002). The persistent high loneliness/high depressive symptoms group showed the greatest risk, with a 97% higher risk of incident osteoporosis (HR = 1.97, 95% CI: 1.37–2.83; *P* < 0.001). Kaplan-Meier curves further supported these findings (log-rank *P* < 0.001; **Figure 4**). The global Schoenfeld residual test was nonsignificant (*P* > 0.05).

### 3.3 Subgroup analyses

**Figure 5:**
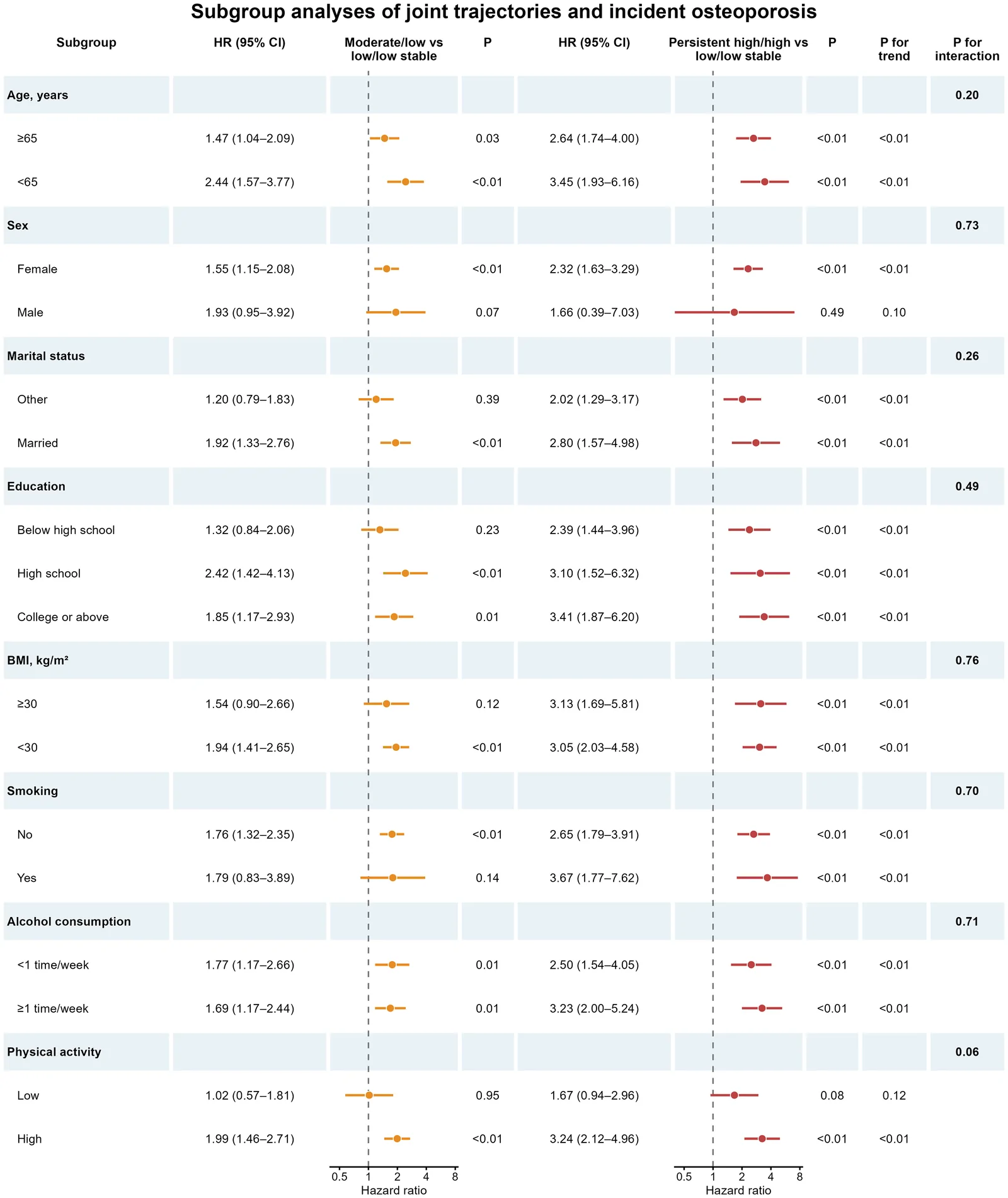
Associations between joint trajectories of loneliness and depressive symptoms and incident osteoporosis across subgroups in ELSA.

We further examined the associations between joint trajectories of loneliness and depressive symptoms and incident osteoporosis across subgroups. The associations remained directionally consistent across strata of age, sex, educational level, marital status, smoking status, alcohol consumption, BMI, and physical activity. The interaction P values were 0.20 for age, 0.73 for sex, 0.26 for marital status, 0.49 for education, 0.76 for BMI, 0.70 for smoking, 0.71 for alcohol consumption, and 0.06 for physical activity; none met the P < 0.05 threshold (**Figure 5**).

### 3.4 Sensitivity analyses

Three sensitivity analyses were conducted to evaluate the robustness of the findings. First, in multivariable logistic regression, the fully adjusted ORs were 1.53 (95% CI: 1.13–2.06; *P* = 0.006) for the moderate loneliness/low depressive symptoms group and 1.97 (95% CI: 1.33–2.92; P < 0.001) for the persistent high loneliness/high depressive symptoms group (**Supplementary Table S6**). Second, after participants who developed osteoporosis during the first follow-up wave were excluded, the corresponding HRs were 1.79 (95% CI: 1.31–2.47; *P* < 0.001) and 1.98 (95% CI: 1.29–3.03; *P* = 0.002), respectively (**Supplementary Table S7**). Third, in the four-class solution, the fully adjusted HRs were 1.58 (95% CI: 1.06–2.36; *P* = 0.026) for the moderate loneliness/moderate depressive symptoms group, 1.77 (95% CI: 1.30–2.41; *P* < 0.001) for the high loneliness/low depressive symptoms group, and 2.05 (95% CI: 1.41–2.98; *P* < 0.001) for the very high loneliness/high depressive symptoms group, compared with the low/low stable group (**Supplementary Table S8**; **Supplementary Figures S1*****–*****S3**). Across all three analyses, the direction and approximate magnitude of the associations were broadly consistent with the primary Cox model.

## 4 Discussion

Using data from a large, nationally representative prospective cohort, we examined joint trajectories of loneliness and depressive symptoms in relation to incident osteoporosis. To our knowledge, this is the first trajectory-based analysis of these longitudinal psychological profiles among middle-aged and older adults. Relative to the low/low stable trajectory, the moderate loneliness/low depressive symptoms and persistent high loneliness/high depressive symptoms trajectories were each associated with a higher hazard of incident osteoporosis. These associations persisted after adjustment for measured confounders, suggesting that the identified trajectories may represent candidate markers of osteoporosis risk. Similar estimates in logistic regression, after excluding early events, and under a four-class solution supported the stability of the findings across alternative analytical specifications. However, these analyses cannot eliminate residual confounding or fully exclude reverse causation.

Our findings align with the broader literature linking poor mental health to a range of chronic diseases and extend current knowledge to skeletal health. Previous studies have shown that depressive symptoms are associated with adverse outcomes, including cardiovascular disease (36), metabolic disorders (37), and cognitive impairment (7). However, evidence regarding bone health has been relatively limited and has largely relied on cross-sectional designs or single-point assessments of psychological status (13, 23, 38). By using longitudinal trajectory modelling, our study highlights the potential relevance of the temporal pattern and persistence of psychological distress to risk assessment. The graded pattern of higher osteoporosis hazards across trajectory groups is consistent with an accumulation of psychological burden over time, although the observational findings do not establish a biological dose-response relationship (39, 40).

Several interrelated biological and behavioral pathways may be relevant to the observed associations between long-term loneliness, depressive symptoms, and osteoporosis risk. As a chronic psychosocial stressor, loneliness may coexist with depression but may also have distinct biological correlates. Previous evidence has associated persistent loneliness and depressive symptoms with activation of the hypothalamic-pituitary-adrenal axis and sympathetic nervous system and with prolonged exposure to stress hormones such as cortisol (41, 42). Such neuroendocrine dysregulation may suppress bone formation, enhance bone resorption, and disrupt bone-remodeling homeostasis (18, 43, 44). Both loneliness and depressive symptoms have also been associated with chronic low-grade inflammation (45, 46). Loneliness has increasingly been characterized as a pro-inflammatory psychosocial state (47, 48), whereas depression is commonly accompanied by higher levels of pro-inflammatory cytokines (49). These inflammatory mediators may promote osteoclast differentiation and activation, thereby accelerating bone resorption and bone loss (20). Long-term loneliness and depression also often coexist with reduced physical activity (50, 51), inadequate nutritional intake (52, 53), and limited outdoor exposure (54, 55). These behaviors are associated with reduced mechanical loading, insufficient intake of bone-related nutrients, and lower vitamin D synthesis and may partly account for the observed associations (56, 57). The proposed biological and behavioral pathways were not measured directly in this study.

The findings from the physical-activity subgroup analysis should be interpreted cautiously. Although the point estimates were larger among participants reporting high versus low physical activity, the interaction test did not provide conclusive evidence of effect modification (P for interaction = 0.06). The observed difference should therefore be considered exploratory and may reflect chance, limited events within strata, measurement error from self-reported activity, residual confounding, or multiple subgroup comparisons. These findings do not demonstrate that physical activity strengthens the association, nor do they imply that physical activity is unimportant for bone health. Larger studies using objective activity measures are needed to determine whether the apparent heterogeneity is reproducible.

The findings have potential clinical and public health relevance but should be interpreted as associative. Psychological well-being may merit consideration alongside established factors during comprehensive risk assessment, without treating the identified trajectories as causal determinants of osteoporosis. In nursing and community settings, brief repeated assessments of loneliness and depressive symptoms may help identify older adults with sustained psychosocial needs; a positive screen should prompt appropriate psychological assessment and referral rather than be interpreted as an osteoporosis diagnosis or treatment criterion. For adults with established bone-health risk factors, nurses can reinforce medication review, falls-risk assessment, appropriate weight-bearing activity, adequate nutrition, and referral for formal fracture-risk or bone-density evaluation. The findings are consistent with integrated models of healthy ageing in which mental-health support and chronic-disease care are addressed together (58). Because the study did not assess interventions, whether reducing loneliness or depressive symptoms changes skeletal outcomes remains unknown.

This study has several strengths, including its prospective design, the use of a large nationally representative sample, repeated assessment of psychological exposures, and trajectory modelling of their joint patterns. Nonetheless, several limitations should be acknowledged. Because the magnitude of each potential bias could not be quantified empirically, the following order reflects their anticipated relevance to internal validity, interpretation, and generalizability. First, residual confounding remains a major concern despite multivariable adjustment. Detailed information on systemic glucocorticoids, bisphosphonates, and other bone-active medications was unavailable, so medication-based exclusion and adjustment were not possible. As an observational study, the analysis therefore cannot establish causality. Second, incident osteoporosis was identified from self-reported physician diagnosis, which may have caused outcome misclassification. Third, complete-case eligibility and loss to follow-up may have introduced selection bias. Fourth, latent-class assignment was probabilistic, and the number and labels of the classes depended on modelling choices. The trajectories were based on the earliest three pre-index assessments and did not capture later psychological changes; a time-varying survival model would address a different, complementary question. Fifth, inflammatory markers, cortisol, and bone-turnover biomarkers were unavailable, so the proposed mechanisms could not be tested directly. Finally, because the study population consisted predominantly of White British adults, the applicability of the findings to other ethnic and cultural populations remains uncertain.

Future studies should seek to replicate these findings in independent cohorts from different countries and populations and incorporate more objective skeletal outcomes, such as longitudinal bone mineral density measurements or clinically confirmed fragility fractures. Studies should also compare trajectory and time-varying exposure models and account explicitly for bone-active medications. In addition, studies integrating biological data, including inflammatory markers and stress hormones, may help clarify the mechanisms linking psychological distress to skeletal deterioration. Ultimately, randomized controlled trials are needed to determine whether interventions aimed at improving mental health or reducing loneliness can slow bone loss or reduce fracture risk, thereby providing stronger evidence for integrated prevention strategies in ageing populations.

## 5 Conclusions

In conclusion, unfavorable long-term trajectories of loneliness and depressive symptoms were associated with a higher risk of incident osteoporosis among MAOAs. These findings identify psychological well-being as a candidate marker for further evaluation in osteoporosis risk assessment but do not establish a causal relationship. Future studies are needed to confirm the associations and determine whether changes in loneliness or depressive symptoms are followed by changes in skeletal health outcomes.

## Data Availability

The original contributions presented in the study are included in the article/**Supplementary Material**, further inquiries can be directed to the corresponding author.
