# Supplementary material for: Joint trajectories of loneliness and depressive symptoms and risk of incident osteoporosis among middle-aged and older adults: a prospective cohort study
Source: Front Endocrinol (Lausanne). 2026 Sep 3;17:1866762. doi: 10.3389/fendo.2026.1866762 (PMC13581791; doi:10.3389/fendo.2026.1866762)
Supplement: Supplementary file 1 [file DataSheet1.pdf]

Supplementary Material

# Joint Trajectories of Loneliness and Depressive Symptoms and Risk of Incident Osteoporosis Among Middle-Aged and Older Adults: A Prospective Cohort Study

Supplementary Table S1. Covariate missingness and operational definitions.

Supplementary Table S2. Fit statistics for candidate GBMTM specifications.

Supplementary Table S3. Classification diagnostics for candidate GBMTM specifications.

Supplementary Table S4. Parameter estimates for candidate GBMTM specifications.

Supplementary Table S5. Classification performance of the selected three-class model.

Supplementary Table S6. Logistic-regression sensitivity analysis of joint trajectories and incident osteoporosis.

Supplementary Table S7. Cox sensitivity analysis excluding osteoporosis events at the first follow-up wave.

Supplementary Table S8. Cox sensitivity analysis using a four-class trajectory solution.

Supplementary Figure S1. Four-class joint trajectories of loneliness and depressive symptoms.

Supplementary Figure S2. Cumulative-incidence curves for osteoporosis in the four-class solution.

Supplementary Figure S3. Subgroup associations in the four-class sensitivity analysis.

## Supplementary Table S1 | Covariate missingness and operational definitions.

| Covariate | Number | Percentage (%) |
|---|---|---|
| BMI | 508 | 12.3 |
| Education | 36 | 8.8 |
| Smoke | 26 | 0.6 |
| Alcohol consumption | 84 | 2 |

Operational definitions: marital status was married versus other (cohabiting, divorced, never married, separated, or widowed). Physical activity was high when moderate- or vigorous-intensity activity was reported at least once per week and low otherwise. Household wealth was classified into sample-specific quintiles (Q1, lowest, to Q5, highest), with Q1 as the reference category; no external fixed cut-offs were used.

**Supplementary Table S2 | Fit statistics for candidate GBMTM specifications.**

Design window and model notation: loneliness was first assessed at Wave 2. Waves 2–4 were therefore the earliest three consecutive assessments, providing the minimum repeated observations for trajectory modelling while retaining the longest subsequent outcome follow-up from the Wave 4 index date through Waves 5–9. M1–M3 are two-class, M4–M6 three-class, and M7–M9 four-class solutions. Within each class count, [11], [22], and [33] denote linear, quadratic, and cubic polynomial orders for both outcomes; thus M4[111][111] is a three-class model with a linear term for loneliness and CES-D-8 in every class. Model selection considered BIC, entropy, APP, OCC, minimum class size, parsimony, and substantive interpretability. BIC became progressively less negative as classes were added, whereas entropy was similar for the three- and four-class linear solutions (0.910 and 0.913). M4 had APPs above 91%, OCC values above 15, and a minimum observed class proportion of 7.63%. M7 also met classification thresholds but primarily subdivided the intermediate pattern into two smaller phenotypes. M4 was therefore retained as the parsimonious primary model, and M7 was evaluated as a sensitivity analysis rather than excluded..

| Model ID | Log likelihood | *BIC* | *AIC* | Entropy |
|---|---|---|---|---|
| M1[11][11] | -42109.659 | -42155.435 | -42120.659 | 0.936 |
| M2[22][22] | -42101.759 | -42164.180 | -42116.759 | 0.936 |
| M3[33][33] | -42101.759 | -42180.826 | -42120.759 | 0.936 |
| M4[111][111] | -40927.969 | -40994.552 | -40943.969 | 0.910 |
| M5[222][222] | -40912.521 | -41004.072 | -40934.521 | 0.911 |
| M6[333][333] | -40912.521 | -41029.041 | -40940.521 | 0.911 |
| M7[1111][1111] | -40302.412 | -40389.803 | -40323.412 | 0.913 |
| M8[2222][2222] | -40287.442 | -40408.124 | -40316.442 | 0.913 |
| M9[3333][3333] | -40287.442 | -40441.416 | -40324.442 | 0.913 |

**Supplementary Table S3 | Classification diagnostics for candidate GBMTM specifications.**

| Model ID | Latent class | OCC | APP (%) | Observed proportion (%) | Model-estimated proportion (%) |
|---|---|---|---|---|---|
| M1[11][11] | Class1 | 19.264 | 98.776 | 80.884 | 80.723 |
| M1[11][11] | Class2 | 92.318 | 95.661 | 19.116 | 19.277 |
| M2[22][22] | Class1 | 18.855 | 98.752 | 80.957 | 80.757 |
| M2[22][22] | Class2 | 94.440 | 95.745 | 19.043 | 19.243 |
| M3[33][33] | Class1 | 18.854 | 98.752 | 80.957 | 80.757 |
| M3[33][33] | Class2 | 94.441 | 95.745 | 19.043 | 19.243 |
| M4[111][111] | Class1 | 36.913 | 91.205 | 21.545 | 21.931 |
| M4[111][111] | Class2 | 15.745 | 97.409 | 70.828 | 70.483 |
| M4[111][111] | Class3 | 196.148 | 94.152 | 7.627 | 7.586 |
| M5[222][222] | Class1 | 15.674 | 97.400 | 70.877 | 70.503 |
| M5[222][222] | Class2 | 37.592 | 91.290 | 21.423 | 21.802 |
| M5[222][222] | Class3 | 217.633 | 94.776 | 7.700 | 7.695 |
| M6[333][333] | Class1 | 15.675 | 97.400 | 70.877 | 70.503 |
| M6[333][333] | Class2 | 37.592 | 91.290 | 21.423 | 21.802 |
| M6[333][333] | Class3 | 217.634 | 94.776 | 7.700 | 7.695 |
| M7[1111][1111] | Class1 | 17.993 | 97.438 | 68.132 | 67.887 |
| M7[1111][1111] | Class2 | 64.573 | 85.271 | 8.088 | 8.228 |
| M7[1111][1111] | Class3 | 49.032 | 90.794 | 16.517 | 16.746 |
| M7[1111][1111] | Class4 | 220.712 | 94.434 | 7.263 | 7.139 |
| M8[2222][2222] | Class1 | 17.424 | 97.348 | 68.181 | 67.808 |
| M8[2222][2222] | Class2 | 67.330 | 85.831 | 8.040 | 8.254 |

| Model ID | Latent class | OCC | APP (%) | Observed proportion (%) | Model-estimated proportion (%) |
|---|---|---|---|---|---|
| M8[2222][2222] | Class3 | 50.576 | 91.032 | 16.468 | 16.716 |
| M8[2222][2222] | Class4 | 244.780 | 95.013 | 7.311 | 7.221 |
| M9[3333][3333] | Class1 | 17.423 | 97.347 | 68.181 | 67.808 |
| M9[3333][3333] | Class2 | 67.330 | 85.831 | 8.040 | 8.254 |
| M9[3333][3333] | Class3 | 50.577 | 91.033 | 16.468 | 16.716 |
| M9[3333][3333] | Class4 | 244.795 | 95.013 | 7.311 | 7.221 |

## Supplementary Table S4 | Parameter estimates for candidate GBMTM specifications.

| Model ID | Traj_Y | Parameter | *Class* | *β* | *SE* | *t* | *P* |
|---|---|---|---|---|---|---|---|
| M1[11][11] | Loneliness scores | Intercept | Class1 | 3.464 | 0.040 | 86.943 | <0.001 |
| M1[11][11] | Loneliness scores | Linear | Class1 | 0.026 | 0.013 | 2.015 | 0.044 |
| M1[11][11] | Loneliness scores | Intercept | Class2 | 5.651 | 0.086 | 65.810 | <0.001 |
| M1[11][11] | Loneliness scores | Linear | Class2 | 0.164 | 0.027 | 5.999 | <0.001 |
| M1[11][11] | CESD scores | Intercept | Class1 | 0.972 | 0.058 | 16.791 | <0.001 |
| M1[11][11] | CESD scores | Linear | Class1 | -0.063 | 0.019 | -3.391 | 0.001 |
| M1[11][11] | CESD scores | Intercept | Class2 | 3.327 | 0.125 | 26.692 | <0.001 |
| M1[11][11] | CESD scores | Linear | Class2 | -0.092 | 0.039 | -2.347 | 0.019 |
| M2[22][22] | Loneliness scores | Intercept | Class1 | 3.534 | 0.187 | 18.880 | <0.001 |
| M2[22][22] | Loneliness scores | Linear | Class1 | -0.024 | 0.132 | -0.184 | 0.854 |
| M2[22][22] | Loneliness scores | Quadratic | Class1 | 0.008 | 0.022 | 0.380 | 0.704 |
| M2[22][22] | Loneliness scores | Intercept | Class2 | 4.198 | 0.398 | 10.557 | <0.001 |
| M2[22][22] | Loneliness scores | Linear | Class2 | 1.211 | 0.281 | 4.305 | <0.001 |
| M2[22][22] | Loneliness scores | Quadratic | Class2 | -0.174 | 0.047 | -3.740 | <0.001 |
| M2[22][22] | CESD scores | Intercept | Class1 | 1.323 | 0.273 | 4.838 | <0.001 |
| M2[22][22] | CESD scores | Linear | Class1 | -0.315 | 0.193 | -1.631 | 0.103 |
| M2[22][22] | CESD scores | Quadratic | Class1 | 0.042 | 0.032 | 1.311 | 0.190 |
| M2[22][22] | CESD scores | Intercept | Class2 | 3.148 | 0.578 | 5.449 | <0.001 |
| M2[22][22] | CESD scores | Linear | Class2 | 0.039 | 0.408 | 0.094 | 0.925 |
| M2[22][22] | CESD scores | Quadratic | Class2 | -0.022 | 0.068 | -0.321 | 0.749 |
| M3[33][33] | Loneliness scores | Intercept | Class1 | 3.324 | 191.837 | 0.017 | 0.986 |
| M3[33][33] | Loneliness scores | Linear | Class1 | 0.204 | 207.823 | 0.001 | 0.999 |
| M3[33][33] | Loneliness scores | Quadratic | Class1 | -0.071 | 71.939 | -0.001 | 0.999 |
| M3[33][33] | Loneliness scores | Cubic | Class1 | 0.009 | 7.993 | 0.001 | 0.999 |
| M3[33][33] | Loneliness scores | Intercept | Class2 | 5.143 | 286.618 | 0.018 | 0.986 |
| M3[33][33] | Loneliness scores | Linear | Class2 | 0.187 | 310.502 | 0.001 | 1.000 |
| M3[33][33] | Loneliness scores | Quadratic | Class2 | 0.180 | 107.482 | 0.002 | 0.999 |
| M3[33][33] | Loneliness scores | Cubic | Class2 | -0.039 | 11.942 | -0.003 | 0.997 |
| M3[33][33] | CESD scores | Intercept | Class1 | 0.623 | 519.569 | 0.001 | 0.999 |
| M3[33][33] | CESD scores | Linear | Class1 | 0.443 | 562.866 | 0.001 | 0.999 |
| M3[33][33] | CESD scores | Quadratic | Class1 | -0.221 | 194.838 | -0.001 | 0.999 |
| M3[33][33] | CESD scores | Cubic | Class1 | 0.029 | 21.649 | 0.001 | 0.999 |
| M3[33][33] | CESD scores | Intercept | Class2 | 2.839 | 929.576 | 0.003 | 0.998 |
| M3[33][33] | CESD scores | Linear | Class2 | 0.373 | 1007.041 | 0.000 | 1.000 |
| M3[33][33] | CESD scores | Quadratic | Class2 | -0.138 | 348.591 | -0.000 | 1.000 |
| M3[33][33] | CESD scores | Cubic | Class2 | 0.013 | 38.732 | 0.000 | 1.000 |
| M4[111][111] | Loneliness scores | Intercept | Class1 | 4.878 | 0.081 | 59.916 | <0.001 |
| M4[111][111] | Loneliness scores | Linear | Class1 | 0.126 | 0.026 | 4.923 | <0.001 |
| M4[111][111] | Loneliness scores | Intercept | Class2 | 3.326 | 0.039 | 85.211 | <0.001 |
| M4[111][111] | Loneliness scores | Linear | Class2 | 0.011 | 0.013 | 0.872 | 0.383 |
| M4[111][111] | Loneliness scores | Intercept | Class3 | 6.214 | 0.127 | 49.078 | <0.001 |
| M4[111][111] | Loneliness scores | Linear | Class3 | 0.225 | 0.040 | 5.561 | <0.001 |
| M4[111][111] | CESD scores | Intercept | Class1 | 1.876 | 0.118 | 15.872 | <0.001 |
| M4[111][111] | CESD scores | Linear | Class1 | -0.054 | 0.037 | -1.462 | 0.144 |
| M4[111][111] | CESD scores | Intercept | Class2 | 0.907 | 0.059 | 15.355 | <0.001 |
| M4[111][111] | CESD scores | Linear | Class2 | -0.064 | 0.019 | -3.379 | 0.001 |

| Model ID | Traj_Y | Parameter | *Class* | *β* | *SE* | *t* | *P* |
|---|---|---|---|---|---|---|---|
| M4[111][111] | CESD scores | Intercept | Class3 | 4.949 | 0.200 | 24.697 | <0.001 |
| M4[111][111] | CESD scores | Linear | Class3 | -0.153 | 0.063 | -2.421 | 0.016 |
| M5[222][222] | Loneliness scores | Intercept | Class1 | 3.527 | 0.183 | 19.310 | <0.001 |
| M5[222][222] | Loneliness scores | Linear | Class1 | -0.134 | 0.129 | -1.036 | 0.300 |
| M5[222][222] | Loneliness scores | Quadratic | Class1 | 0.024 | 0.021 | 1.127 | 0.260 |
| M5[222][222] | Loneliness scores | Intercept | Class2 | 3.926 | 0.363 | 10.826 | <0.001 |
| M5[222][222] | Loneliness scores | Linear | Class2 | 0.813 | 0.257 | 3.169 | 0.002 |
| M5[222][222] | Loneliness scores | Quadratic | Class2 | -0.115 | 0.043 | -2.700 | 0.007 |
| M5[222][222] | Loneliness scores | Intercept | Class3 | 4.149 | 0.573 | 7.237 | <0.001 |
| M5[222][222] | Loneliness scores | Linear | Class3 | 1.694 | 0.403 | 4.200 | <0.001 |
| M5[222][222] | Loneliness scores | Quadratic | Class3 | -0.245 | 0.067 | -3.658 | <0.001 |
| M5[222][222] | CESD scores | Intercept | Class1 | 1.261 | 0.279 | 4.523 | <0.001 |
| M5[222][222] | CESD scores | Linear | Class1 | -0.317 | 0.197 | -1.610 | 0.107 |
| M5[222][222] | CESD scores | Quadratic | Class1 | 0.042 | 0.033 | 1.292 | 0.196 |
| M5[222][222] | CESD scores | Intercept | Class2 | 2.596 | 0.551 | 4.712 | <0.001 |
| M5[222][222] | CESD scores | Linear | Class2 | -0.584 | 0.391 | -1.492 | 0.136 |
| M5[222][222] | CESD scores | Quadratic | Class2 | 0.088 | 0.065 | 1.358 | 0.174 |
| M5[222][222] | CESD scores | Intercept | Class3 | 2.852 | 0.934 | 3.055 | 0.002 |
| M5[222][222] | CESD scores | Linear | Class3 | 1.352 | 0.660 | 2.049 | 0.040 |
| M5[222][222] | CESD scores | Quadratic | Class3 | -0.251 | 0.109 | -2.291 | 0.022 |
| M6[333][333] | Loneliness scores | Intercept | Class1 | 3.057 | 601.377 | 0.005 | 0.996 |
| M6[333][333] | Loneliness scores | Linear | Class1 | 0.375 | 651.490 | 0.001 | 1.000 |
| M6[333][333] | Loneliness scores | Quadratic | Class1 | -0.152 | 225.517 | -0.001 | 0.999 |
| M6[333][333] | Loneliness scores | Cubic | Class1 | 0.020 | 25.057 | 0.001 | 0.999 |
| M6[333][333] | Loneliness scores | Intercept | Class2 | 4.375 | 214.003 | 0.020 | 0.984 |
| M6[333][333] | Loneliness scores | Linear | Class2 | 0.327 | 231.839 | 0.001 | 0.999 |
| M6[333][333] | Loneliness scores | Quadratic | Class2 | 0.054 | 80.252 | 0.001 | 0.999 |
| M6[333][333] | Loneliness scores | Cubic | Class2 | -0.019 | 8.917 | -0.002 | 0.998 |
| M6[333][333] | Loneliness scores | Intercept | Class3 | 5.561 | 4515.028 | 0.001 | 0.999 |
| M6[333][333] | Loneliness scores | Linear | Class3 | 0.165 | 4891.293 | 0.000 | 1.000 |
| M6[333][333] | Loneliness scores | Quadratic | Class3 | 0.285 | 1693.139 | 0.000 | 1.000 |
| M6[333][333] | Loneliness scores | Cubic | Class3 | -0.059 | 188.126 | -0.000 | 1.000 |
| M6[333][333] | CESD scores | Intercept | Class1 | 0.335 | 307.698 | 0.001 | 0.999 |
| M6[333][333] | CESD scores | Linear | Class1 | 0.685 | 333.340 | 0.002 | 0.998 |
| M6[333][333] | CESD scores | Quadratic | Class1 | -0.305 | 115.387 | -0.003 | 0.998 |
| M6[333][333] | CESD scores | Cubic | Class1 | 0.039 | 12.821 | 0.003 | 0.998 |
| M6[333][333] | CESD scores | Intercept | Class2 | 1.715 | 7617.815 | 0.000 | 1.000 |
| M6[333][333] | CESD scores | Linear | Class2 | 0.371 | 8252.631 | 0.000 | 1.000 |
| M6[333][333] | CESD scores | Quadratic | Class2 | -0.242 | 2856.680 | -0.000 | 1.000 |
| M6[333][333] | CESD scores | Cubic | Class2 | 0.037 | 317.409 | 0.000 | 1.000 |
| M6[333][333] | CESD scores | Intercept | Class3 | 3.573 | 1877.356 | 0.002 | 0.998 |
| M6[333][333] | CESD scores | Linear | Class3 | 0.571 | 2033.802 | 0.000 | 1.000 |
| M6[333][333] | CESD scores | Quadratic | Class3 | 0.020 | 704.008 | 0.000 | 1.000 |
| M6[333][333] | CESD scores | Cubic | Class3 | -0.030 | 78.223 | -0.000 | 1.000 |
| M7[1111][1111] | Loneliness scores | Intercept | Class1 | 3.328 | 0.039 | 85.348 | <0.001 |
| M7[1111][1111] | Loneliness scores | Linear | Class1 | 0.010 | 0.013 | 0.789 | 0.430 |
| M7[1111][1111] | Loneliness scores | Intercept | Class2 | 4.246 | 0.149 | 28.454 | <0.001 |

| Model ID | Traj_Y | Parameter | *Class* | ***β*** | ***SE*** | ***t*** | ***P*** |
|---|---|---|---|---|---|---|---|
| M7[1111][1111] | Loneliness scores | Linear | Class2 | -0.013 | 0.046 | -0.276 | 0.783 |
| M7[1111][1111] | Loneliness scores | Intercept | Class3 | 4.984 | 0.096 | 51.908 | <0.001 |
| M7[1111][1111] | Loneliness scores | Linear | Class3 | 0.169 | 0.030 | 5.645 | <0.001 |
| M7[1111][1111] | Loneliness scores | Intercept | Class4 | 6.195 | 0.126 | 49.250 | <0.001 |
| M7[1111][1111] | Loneliness scores | Linear | Class4 | 0.256 | 0.041 | 6.324 | <0.001 |
| M7[1111][1111] | CESD scores | Intercept | Class1 | 0.792 | 0.056 | 14.138 | <0.001 |
| M7[1111][1111] | CESD scores | Linear | Class1 | -0.063 | 0.018 | -3.467 | 0.001 |
| M7[1111][1111] | CESD scores | Intercept | Class2 | 3.959 | 0.272 | 14.544 | <0.001 |
| M7[1111][1111] | CESD scores | Linear | Class2 | -0.220 | 0.086 | -2.550 | 0.011 |
| M7[1111][1111] | CESD scores | Intercept | Class3 | 1.286 | 0.130 | 9.858 | <0.001 |
| M7[1111][1111] | CESD scores | Linear | Class3 | 0.005 | 0.042 | 0.112 | 0.911 |
| M7[1111][1111] | CESD scores | Intercept | Class4 | 4.869 | 0.186 | 26.139 | <0.001 |
| M7[1111][1111] | CESD scores | Linear | Class4 | -0.124 | 0.059 | -2.111 | 0.035 |
| M8[2222][2222] | Loneliness scores | Intercept | Class1 | 3.458 | 0.183 | 18.866 | <0.001 |
| M8[2222][2222] | Loneliness scores | Linear | Class1 | -0.084 | 0.130 | -0.648 | 0.517 |
| M8[2222][2222] | Loneliness scores | Quadratic | Class1 | 0.016 | 0.021 | 0.728 | 0.466 |
| M8[2222][2222] | Loneliness scores | Intercept | Class2 | 4.166 | 0.638 | 6.526 | <0.001 |
| M8[2222][2222] | Loneliness scores | Linear | Class2 | 0.031 | 0.455 | 0.067 | 0.946 |
| M8[2222][2222] | Loneliness scores | Quadratic | Class2 | -0.007 | 0.076 | -0.092 | 0.926 |
| M8[2222][2222] | Loneliness scores | Intercept | Class3 | 3.962 | 0.419 | 9.452 | <0.001 |
| M8[2222][2222] | Loneliness scores | Linear | Class3 | 0.909 | 0.296 | 3.070 | 0.002 |
| M8[2222][2222] | Loneliness scores | Quadratic | Class3 | -0.124 | 0.049 | -2.523 | 0.012 |
| M8[2222][2222] | Loneliness scores | Intercept | Class4 | 4.303 | 0.586 | 7.338 | <0.001 |
| M8[2222][2222] | Loneliness scores | Linear | Class4 | 1.605 | 0.414 | 3.877 | <0.001 |
| M8[2222][2222] | Loneliness scores | Quadratic | Class4 | -0.224 | 0.069 | -3.268 | 0.001 |
| M8[2222][2222] | CESD scores | Intercept | Class1 | 0.941 | 0.262 | 3.595 | <0.001 |
| M8[2222][2222] | CESD scores | Linear | Class1 | -0.171 | 0.185 | -0.925 | 0.355 |
| M8[2222][2222] | CESD scores | Quadratic | Class1 | 0.018 | 0.031 | 0.587 | 0.557 |
| M8[2222][2222] | CESD scores | Intercept | Class2 | 5.095 | 1.031 | 4.940 | <0.001 |
| M8[2222][2222] | CESD scores | Linear | Class2 | -1.058 | 0.726 | -1.456 | 0.145 |
| M8[2222][2222] | CESD scores | Quadratic | Class2 | 0.140 | 0.120 | 1.169 | 0.242 |
| M8[2222][2222] | CESD scores | Intercept | Class3 | 2.480 | 0.583 | 4.252 | <0.001 |
| M8[2222][2222] | CESD scores | Linear | Class3 | -0.855 | 0.410 | -2.086 | 0.037 |
| M8[2222][2222] | CESD scores | Quadratic | Class3 | 0.143 | 0.068 | 2.110 | 0.035 |
| M8[2222][2222] | CESD scores | Intercept | Class4 | 2.786 | 0.869 | 3.205 | 0.001 |
| M8[2222][2222] | CESD scores | Linear | Class4 | 1.372 | 0.615 | 2.232 | 0.026 |
| M8[2222][2222] | CESD scores | Quadratic | Class4 | -0.249 | 0.102 | -2.443 | 0.015 |
| M9[3333][3333] | Loneliness scores | Intercept | Class1 | 2.918 | 566.075 | 0.005 | 0.996 |
| M9[3333][3333] | Loneliness scores | Linear | Class1 | 0.501 | 613.243 | 0.001 | 0.999 |
| M9[3333][3333] | Loneliness scores | Quadratic | Class1 | -0.187 | 212.279 | -0.001 | 0.999 |
| M9[3333][3333] | Loneliness scores | Cubic | Class1 | 0.023 | 23.586 | 0.001 | 0.999 |
| M9[3333][3333] | Loneliness scores | Intercept | Class2 | 3.878 | 2018.468 | 0.002 | 0.998 |
| M9[3333][3333] | Loneliness scores | Linear | Class2 | 0.343 | 2186.702 | 0.000 | 1.000 |
| M9[3333][3333] | Loneliness scores | Quadratic | Class2 | -0.115 | 756.921 | -0.000 | 1.000 |
| M9[3333][3333] | Loneliness scores | Cubic | Class2 | 0.012 | 84.103 | 0.000 | 1.000 |
| M9[3333][3333] | Loneliness scores | Intercept | Class3 | 4.692 | 582.659 | 0.008 | 0.994 |
| M9[3333][3333] | Loneliness scores | Linear | Class3 | 0.118 | 631.215 | 0.000 | 1.000 |

| **Model ID** | **Traj_Y** | **Parameter** | ***Class*** | ***β*** | ***SE*** | ***t*** | ***P*** |
|---|---|---|---|---|---|---|---|
| M9[3333][3333] | Loneliness scores | Quadratic | Class3 | 0.150 | 218.498 | 0.001 | 0.999 |
| M9[3333][3333] | Loneliness scores | Cubic | Class3 | -0.030 | 24.277 | -0.001 | 0.999 |
| M9[3333][3333] | Loneliness scores | Intercept | Class4 | 5.728 | 4506.173 | 0.001 | 0.999 |
| M9[3333][3333] | Loneliness scores | Linear | Class4 | 0.060 | 4881.699 | 0.000 | 1.000 |
| M9[3333][3333] | Loneliness scores | Quadratic | Class4 | 0.310 | 1689.814 | 0.000 | 1.000 |
| M9[3333][3333] | Loneliness scores | Cubic | Class4 | -0.059 | 187.758 | -0.000 | 1.000 |
| M9[3333][3333] | CESD scores | Intercept | Class1 | 0.076 | 166.334 | 0.000 | 1.000 |
| M9[3333][3333] | CESD scores | Linear | Class1 | 0.766 | 180.195 | 0.004 | 0.997 |
| M9[3333][3333] | CESD scores | Quadratic | Class1 | -0.306 | 62.375 | -0.005 | 0.996 |
| M9[3333][3333] | CESD scores | Cubic | Class1 | 0.036 | 6.931 | 0.005 | 0.996 |
| M9[3333][3333] | CESD scores | Intercept | Class2 | 2.604 | 1806.554 | 0.001 | 0.999 |
| M9[3333][3333] | CESD scores | Linear | Class2 | 1.641 | 1957.068 | 0.001 | 0.999 |
| M9[3333][3333] | CESD scores | Quadratic | Class2 | -0.794 | 677.457 | -0.001 | 0.999 |
| M9[3333][3333] | CESD scores | Cubic | Class2 | 0.104 | 75.273 | 0.001 | 0.999 |
| M9[3333][3333] | CESD scores | Intercept | Class3 | 1.814 | 676.720 | 0.003 | 0.998 |
| M9[3333][3333] | CESD scores | Linear | Class3 | -0.134 | 733.117 | -0.000 | 1.000 |
| M9[3333][3333] | CESD scores | Quadratic | Class3 | -0.107 | 253.770 | -0.000 | 1.000 |
| M9[3333][3333] | CESD scores | Cubic | Class3 | 0.028 | 28.197 | 0.001 | 0.999 |
| M9[3333][3333] | CESD scores | Intercept | Class4 | 3.695 | 7564.994 | 0.000 | 1.000 |
| M9[3333][3333] | CESD scores | Linear | Class4 | 0.387 | 8195.411 | 0.000 | 1.000 |
| M9[3333][3333] | CESD scores | Quadratic | Class4 | 0.092 | 2836.873 | 0.000 | 1.000 |
| M9[3333][3333] | CESD scores | Cubic | Class4 | -0.038 | 315.208 | -0.000 | 1.000 |

## Supplementary Table S5 | Classification performance of the selected three-class linear GBMTM (M4).

| Latent class | Descriptive trajectory label | Shape | APP (%) | n | Observed proportion (%) | Model-estimated proportion (%) | OCC | Entropy |
|---|---|---|---|---|---|---|---|---|
| Class 2 | Low/low stable | Linear | 97.409 | 2,916 | 70.828 | 70.483 | 15.745 | 0.910 |
| Class 1 | Moderate loneliness/low depressive symptoms | Linear | 91.205 | 887 | 21.545 | 21.931 | 36.913 | |
| Class 3 | Persistent high loneliness/high depressive symptoms | Linear | 94.152 | 314 | 7.627 | 7.586 | 196.148 | |

APP, average posterior probability; OCC, odds of correct classification.

**Supplementary Table S6 | Logistic-regression sensitivity analysis of joint trajectories and incident osteoporosis.**

| Joint trajectory | Model 1 OR (95% CI) | *P* | Model 2 OR (95% CI) | *P* | Model 3 OR (95% CI) | *P* |
|---|---|---|---|---|---|---|
| Low/low stable | 1.00 (reference) | | 1.00 (reference) | | 1.00 (reference) | |
| Moderate/low | 1.72 (1.29–2.28) | <0.001 | 1.48 (1.10–2.00) | 0.009 | 1.53 (1.13–2.06) | 0.006 |
| Persistent high/high | 2.83 (1.97–4.05) | <0.001 | 1.97 (1.34–2.90) | <0.001 | 1.97 (1.33–2.92) | <0.001 |

OR, odds ratio; CI, confidence interval.

Model 1: unadjusted. Model 2: adjusted for age, sex, ethnicity, marital status, education, and household wealth. Model 3: additionally adjusted for BMI, diabetes, hyperlipidaemia, cancer, hypertension, current smoking, alcohol consumption, and physical activity.

**Supplementary Table S7 | Cox sensitivity analysis after excluding osteoporosis events at the first follow-up wave.**

| Joint trajectory | Model 1 HR (95% CI) | *P* | Model 2 HR (95% CI) | *P* | Model 3 HR (95% CI) | *P* |
|---|---|---|---|---|---|---|
| Low/low stable | 1.00 (reference) | | 1.00 (reference) | | 1.00 (reference) | |
| Moderate/low | 2.00 (1.47–2.70) | <0.001 | 1.73 (1.26–2.37) | <0.001 | 1.79 (1.31–2.47) | <0.001 |
| Persistent high/high | 2.91 (1.96–4.32) | <0.001 | 2.01 (1.32–3.05) | 0.001 | 1.98 (1.29–3.03) | 0.002 |

HR, hazard ratio; CI, confidence interval.

Model 1: unadjusted. Model 2: adjusted for age, sex, ethnicity, marital status, education, and household wealth. Model 3: additionally adjusted for BMI, diabetes, hyperlipidaemia, cancer, hypertension, current smoking, alcohol consumption, and physical activity.

## Supplementary Table S8 | Cox sensitivity analysis using the four-class linear trajectory solution.

| Joint trajectory | Model 1 HR (95% CI) | *P* | Model 2 HR (95% CI) | *P* | Model 3 HR (95% CI) | *P* |
|---|---|---|---|---|---|---|
| Low/low stable | 1.00 (reference) | | 1.00 (reference) | | 1.00 (reference) | |
| Moderate loneliness/moderate depressive symptoms | 1.85 (1.24–2.74) | 0.002 | 1.56 (1.05–2.32) | 0.029 | 1.58 (1.06–2.36) | 0.026 |
| High loneliness/low depressive symptoms | 1.97 (1.46–2.65) | <0.001 | 1.69 (1.24–2.30) | <0.001 | 1.77 (1.30–2.41) | <0.001 |
| Very high loneliness/high depressive symptoms | 3.13 (2.21–4.43) | <0.001 | 2.08 (1.44–3.01) | <0.001 | 2.05 (1.41–2.98) | <0.001 |

HR, hazard ratio; CI, confidence interval.

Model 1: unadjusted. Model 2: adjusted for age, sex, ethnicity, marital status, education, and household wealth. Model 3: additionally adjusted for BMI, diabetes, hyperlipidaemia, cancer, hypertension, current smoking, alcohol consumption, and physical activity.

**Supplementary Figure S1 |** Four-class joint trajectories of loneliness and depressive symptoms. The four fitted classes were low loneliness/low depressive symptoms stable (68.13%; n = 2,805), moderate loneliness/moderate depressive symptoms (8.09%; n = 333), high loneliness/low depressive symptoms (16.52%; n = 680), and very high loneliness/high depressive symptoms (7.26%; n = 299). Solid lines and points show model-predicted means across ELSA Waves 2-4; shaded bands denote 95% confidence intervals. The upper panels show UCLA-3 loneliness scores and the lower panels show CES-D-8 scores.

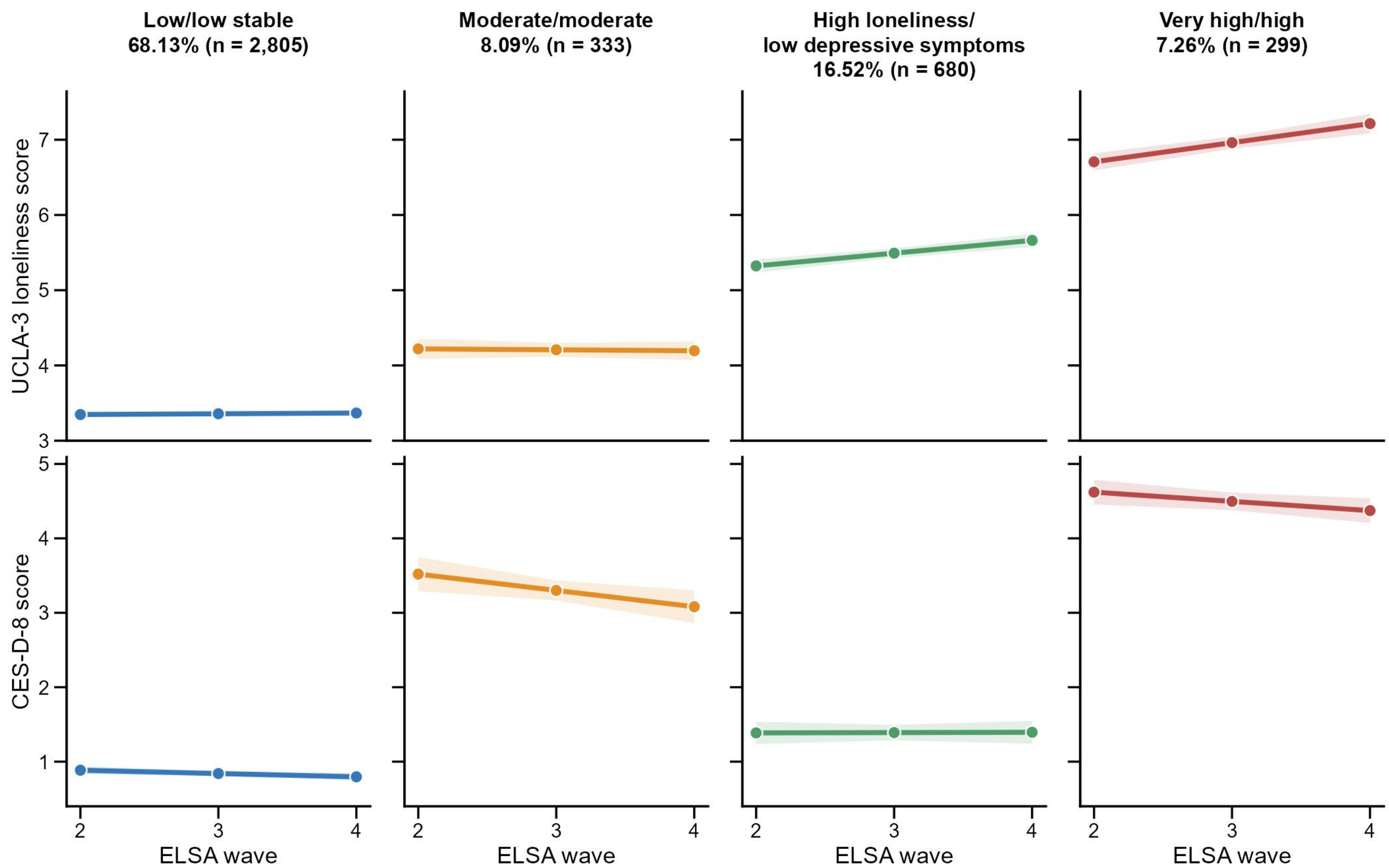

**Supplementary Figure S2 |** Cumulative incidence of osteoporosis in the four-class trajectory solution. Curves show 1 minus the Kaplan-Meier survival estimate from the Wave 4 index date through 100 months, with numbers at risk shown below. The P value is from a two-sided log-rank test. Colours and trajectory labels correspond to Supplementary Figure S1.

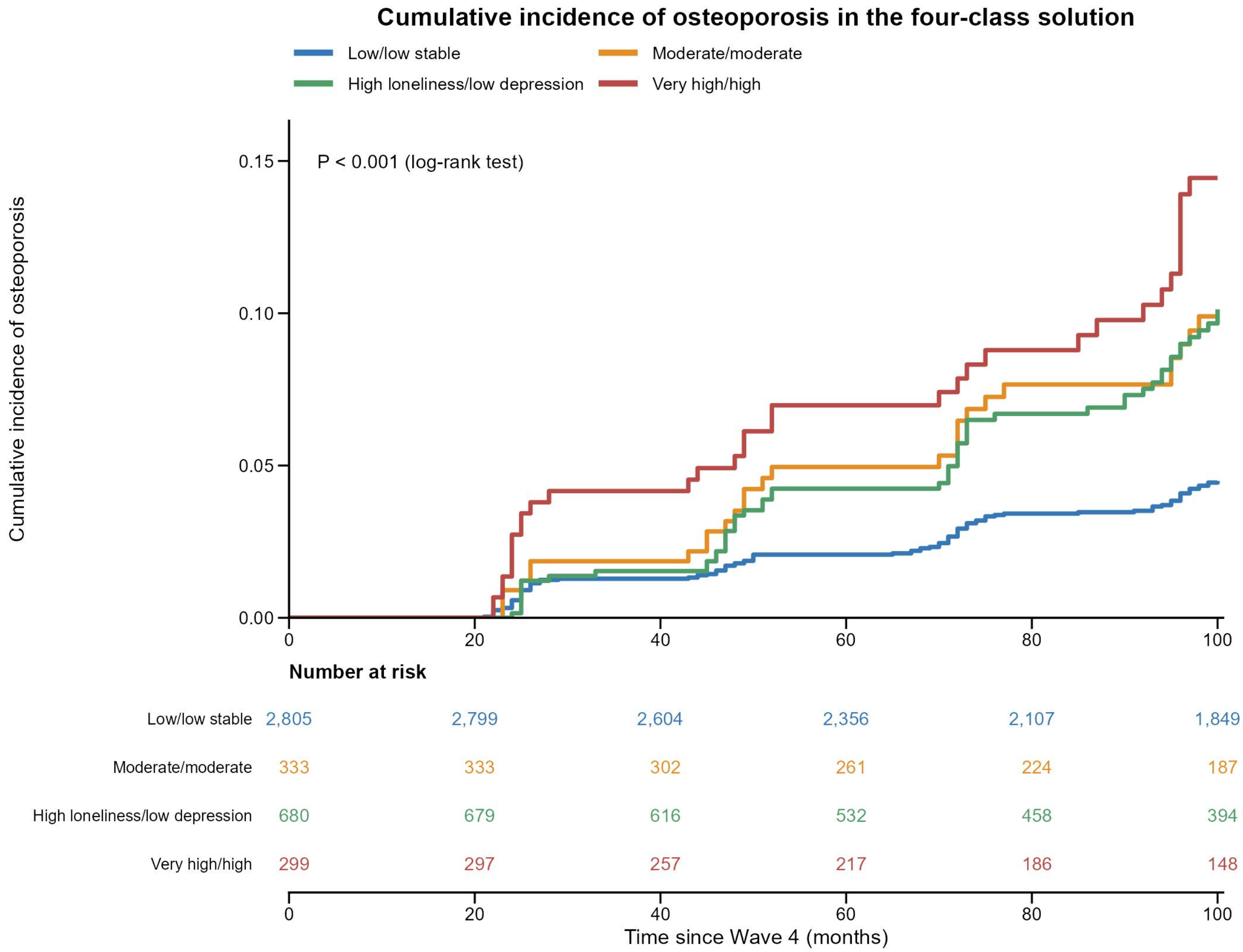

**Supplementary Figure S3 |** Subgroup associations between the four-class joint trajectories and incident osteoporosis. Within each subgroup, unadjusted Cox proportional-hazards models estimated hazard ratios (HRs) and 95% confidence intervals (CIs) for Group 2, Group 3, and Group 4 versus Group 1. Group 1 denotes low loneliness/low depressive symptoms stable; Group 2, moderate loneliness/moderate depressive symptoms; Group 3, high loneliness/low depressive symptoms; and Group 4, very high loneliness/high depressive symptoms. P for trend was derived by coding Groups 1-4 as 0-3, respectively. P for interaction was obtained from likelihood-ratio tests comparing models with and without the trajectory-by-subgroup interaction term.

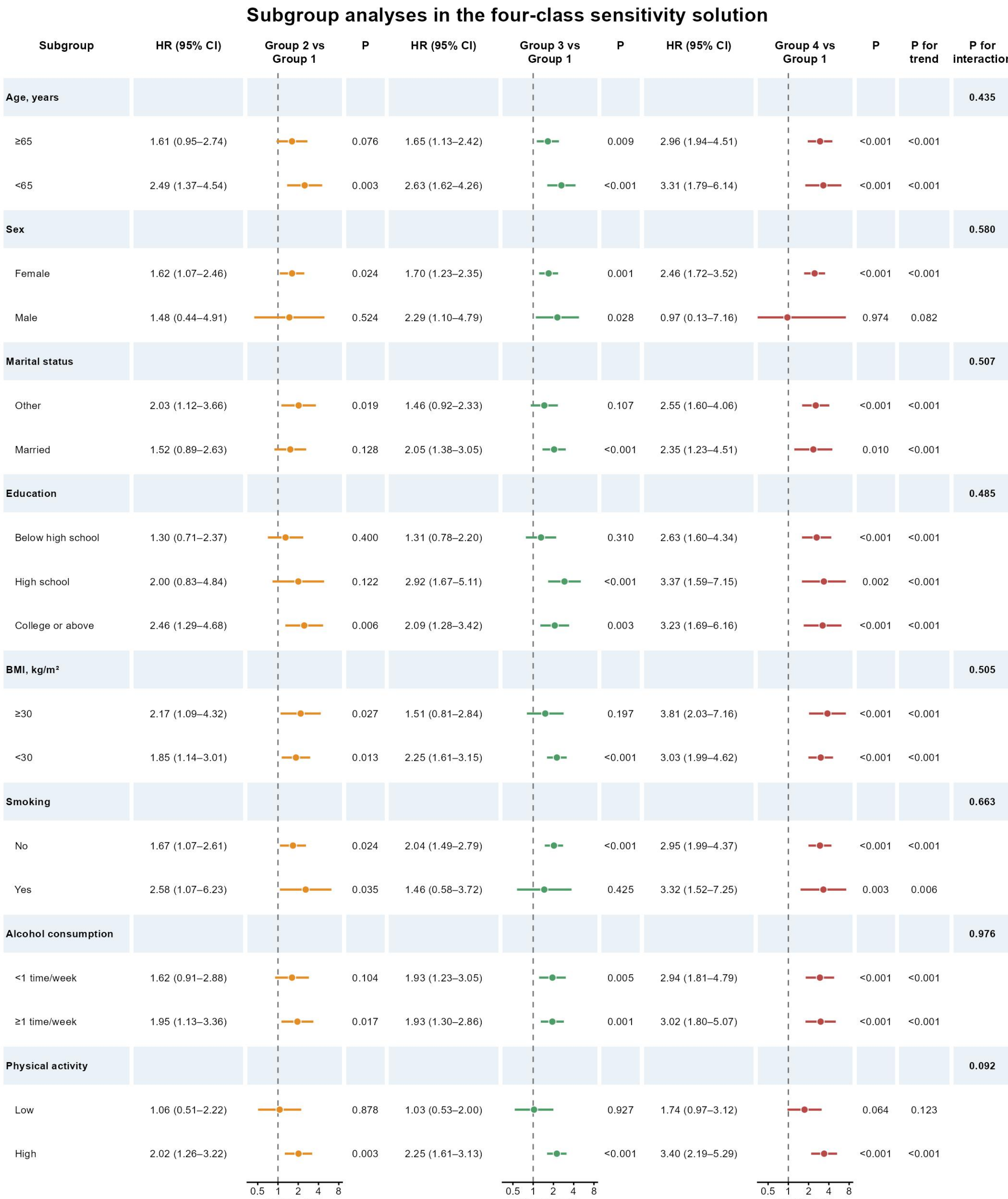

**Subgroup analyses in the four-class sensitivity solution**

| Subgroup | HR (95% CI) | Group 2 vs Group 1 | P | HR (95% CI) | Group 3 vs Group 1 | P | HR (95% CI) | Group 4 vs Group 1 | P | P for trend | P for interaction |
|---|---|---|---|---|---|---|---|---|---|---|---|
| **Age, years** | | | | | | | | | | | **0.435** |
| ≥65 | 1.61 (0.95–2.74) | | 0.076 | 1.65 (1.13–2.42) | | 0.009 | 2.96 (1.94–4.51) | | <0.001 | <0.001 | |
| <65 | 2.49 (1.37–4.54) | | 0.003 | 2.63 (1.62–4.26) | | <0.001 | 3.31 (1.79–6.14) | | <0.001 | <0.001 | |
| **Sex** | | | | | | | | | | | **0.580** |
| Female | 1.62 (1.07–2.46) | | 0.024 | 1.70 (1.23–2.35) | | 0.001 | 2.46 (1.72–3.52) | | <0.001 | <0.001 | |
| Male | 1.48 (0.44–4.91) | | 0.524 | 2.29 (1.10–4.79) | | 0.028 | 0.97 (0.13–7.16) | | 0.974 | 0.082 | |
| **Marital status** | | | | | | | | | | | **0.507** |
| Other | 2.03 (1.12–3.66) | | 0.019 | 1.46 (0.92–2.33) | | 0.107 | 2.55 (1.60–4.06) | | <0.001 | <0.001 | |
| Married | 1.52 (0.89–2.63) | | 0.128 | 2.05 (1.38–3.05) | | <0.001 | 2.35 (1.23–4.51) | | 0.010 | <0.001 | |
| **Education** | | | | | | | | | | | **0.485** |
| Below high school | 1.30 (0.71–2.37) | | 0.400 | 1.31 (0.78–2.20) | | 0.310 | 2.63 (1.60–4.34) | | <0.001 | <0.001 | |
| High school | 2.00 (0.83–4.84) | | 0.122 | 2.92 (1.67–5.11) | | <0.001 | 3.37 (1.59–7.15) | | 0.002 | <0.001 | |
| College or above | 2.46 (1.29–4.68) | | 0.006 | 2.09 (1.28–3.42) | | 0.003 | 3.23 (1.69–6.16) | | <0.001 | <0.001 | |
| **BMI, kg/m²** | | | | | | | | | | | **0.505** |
| ≥30 | 2.17 (1.09–4.32) | | 0.027 | 1.51 (0.81–2.84) | | 0.197 | 3.81 (2.03–7.16) | | <0.001 | <0.001 | |
| <30 | 1.85 (1.14–3.01) | | 0.013 | 2.25 (1.61–3.15) | | <0.001 | 3.03 (1.99–4.62) | | <0.001 | <0.001 | |
| **Smoking** | | | | | | | | | | | **0.663** |
| No | 1.67 (1.07–2.61) | | 0.024 | 2.04 (1.49–2.79) | | <0.001 | 2.95 (1.99–4.37) | | <0.001 | <0.001 | |
| Yes | 2.58 (1.07–6.23) | | 0.035 | 1.46 (0.58–3.72) | | 0.425 | 3.32 (1.52–7.25) | | 0.003 | 0.006 | |
| **Alcohol consumption** | | | | | | | | | | | **0.976** |
| <1 time/week | 1.62 (0.91–2.88) | | 0.104 | 1.93 (1.23–3.05) | | 0.005 | 2.94 (1.81–4.79) | | <0.001 | <0.001 | |
| ≥1 time/week | 1.95 (1.13–3.36) | | 0.017 | 1.93 (1.30–2.86) | | 0.001 | 3.02 (1.80–5.07) | | <0.001 | <0.001 | |
| **Physical activity** | | | | | | | | | | | **0.092** |
| Low | 1.06 (0.51–2.22) | | 0.878 | 1.03 (0.53–2.00) | | 0.927 | 1.74 (0.97–3.12) | | 0.064 | 0.123 | |
| High | 2.02 (1.26–3.22) | | 0.003 | 2.25 (1.61–3.13) | | <0.001 | 3.40 (2.19–5.29) | | <0.001 | <0.001 | |
